# Weekly multimodal MRI follow-up of two multiple sclerosis active lesions presenting a transient decrease in ADC

**DOI:** 10.1002/brb3.307

**Published:** 2015-01-16

**Authors:** Salem Hannoun, Jean-Amédée Roch, Francoise Durand-Dubief, Sandra Vukusic, Dominique Sappey-Marinier, Charles RG Guttmann, Francois Cotton

**Affiliations:** 1 CREATIS – CNRS UMR 5220 & INSERM U1044, Université Claude Bernard-Lyon1Villeurbanne, France; 2 Service de Neurologie A & EDMUS Coordinating Center for Multiple Sclerosis, Hôpital Neurologique Pierre WertheimerBron, France; 3 Service de Radiologie, Centre Hospitalier Lyon-Sud, Hospices Civils de LyonPierre-Bénite, France; 4 CERMEP – Imagerie du Vivant, Groupement Hospitalier EstBron, France; 5 Center for Neurological Imaging, Brigham and Women's HospitalBoston, Massachusetts

**Keywords:** Apparent diffusion coefficient, diffusion tensor imaging, lesions, multiple sclerosis, perfusion, relative cerebral blood volume

## Abstract

**Background and purpose:**

Blood-brain barrier disruption during the earliest phases of lesion formation in multiple sclerosis (MS) patients is commonly ascribed to perivenular inflammatory activity and is usually accompanied by increased diffusivity. Reduced diffusivity has also been shown in active lesions, albeit less frequently. This study aimed to characterize the development and natural history of contrast-enhanced lesions by weekly following five relapsing remitting (RR) MS patients.

**Materials and methods:**

Diffusion tensor imaging (DTI), perfusion imaging, FLAIR and contrast-enhanced 3D T1-weighted MR, were weekly performed on five untreated patients recently diagnosed with RR MS.

**Results:**

All five patients showed significant increases of the apparent diffusion coefficient (ADC) in the lesions compared to the first time point. One of the five patients presented 98 active lesions on ADC maps among which 36 had a volume larger than 10 mm^3^. In two of these lesions, a 1 week transient decrease in ADC was detected at the time of the first gadolinium enhancement. Also, the perfusion analysis showed a concomitant increase in the relative cerebral blood volume.

**Conclusions:**

The infrequency detection of such ADC decrease in a new lesion is probably due to its very short duration. This observation may be consistent with a hyper-acute inflammatory stage concomitant with an increased reactional perfusion.

## Introduction

Blood-brain barrier (BBB) disruption during the earliest phases of brain lesion formation in multiple sclerosis (MS) patients is commonly ascribed to perivenular inflammatory activity (Adams 1975; Vos et al. 2005). This interpretation is supported by the histopathological observation of perivenular cuffs composed of T-cells. Lucchinetti et al. (2000) and Lassman (2003) have described a subpopulation of MS lesions (Type III) that demonstrate histological features consistent with hypoxic damage.

Most studies have reported increased values of the apparent diffusion coefficient (ADC) in acute MS lesions, consistent with inflammation-related extracellular vasogenic edema (Rocca et al. 2000; Castriota-Scanderbeg et al. 2002; Wuerfel et al. 2004). A less common finding in the early stages of lesion formation is that of reduced diffusion, with decreased ADC, possibly reflecting additional pathophysiological mechanisms, such as cytotoxic edema or localized hypercellularity (Bathia and Garg 2007). Decreased diffusion has also been observed in other demyelinating diseases, such as acute disseminated encephalomyelitis and Balo's sclerosis, but rarely in MS (Rocca et al. 2000) where it has been primarily reported at the outer rim of active lesions (Tievsky et al. 1999). In order to better understand the development and natural history of contrast-enhanced lesions, we performed a weekly MRI follow-up in five relapsing remitting (RR) MS patients. In this paper, we describe the weekly evolution of two newly appearing MS lesions demonstrating transient reduced diffusion on ADC maps.

## Methods

### Subject

Five MS patients underwent a weekly MRI follow-up for 2 months (eight MRI examinations). To be included in this study, patients had to be untreated for at least one year and have at least one active gadolinium-enhanced lesion during the 6 months preceding study enrollment. Patients were excluded if they presented with other brain comorbidities (microangiopathy, ischemic stroke), had contraindications to undergo MRI (pace-maker, cardiac vasculopathy, claustrophobia, allergy to contrast agent, and pregnancy) or renal insufficiency with low creatinine clearance. This longitudinal prospective study (ClinicalTrials.gov Identifier: NCT00861172) was approved by the local ethics committee (CPP Lyon Sud-Est IV) and the French Health Products Safety Agency (AFSSAPS). All patients signed an informed consent form approved by the ethics committee.

### Image acquisition

This work is part of a multimodal imaging study comprising anatomical, metabolic, diffusion tensor and perfusion MRI. MRI acquisitions were performed on a 3T MRI (Achieva 3T, Philips Medical Systems, Best, the Netherlands) with a 16-channel head coil, on the same day, at the same time (every Tuesday at noon), for all patients between March and December 2009. All sequences were acquired in the same axial plane defined by the anterior and posterior commissures (AC-PC).

The conventional MRI protocol included a sagittal 3D fluid attenuated inversion recovery (FLAIR) sequence (TE/TR/TI: 356/8000/2400 ms; slice thickness: 1.2 mm; acquisition matrix: 228 * 226; reconstruction matrix of 576 * 576 for a 250 mm field-of-view (FOV) yielding a nominal in-plane pixel size of 0.434 * 0.434 mm), and an axial 3D T1 postgadolinium (Gd) contrast turbo field echo sequence, (TR/TE: 6.7/3 ms; slice thickness: 0.9 mm; acquisition matrix: 268 * 211; reconstruction matrix: 512 * 512; FOV: 240 mm; yielding a nominal in plane pixel size of 0.469 * 0.469 mm). A standard dose of 0.1 mmol/kg Gadobutrol (Gadovist ©, Berlin, Germany) was administered during each MRI with a delay of 60 sec between injection and the 3D T1 acquisition.

Diffusion Tensor Imaging (DTI) protocol consisted in the acquisition of a 2D echo-planar imaging (EPI) sequence before gadolinium injection (TR/TE/TI: 8.21/106/2.5 ms; slice thickness: 2 mm; FOV: 224 * 224 * 120 mm; nominal isotropic voxel dimension was 2 * 2 * 2 mm^3^ and 0.875 * 0.875 * 2 mm^3^ after reconstruction. Diffusion gradients were applied along 32 noncollinear directions with a b factor of 1000 s/mm^2^.

MR perfusion images were acquired using a dynamic susceptibility contrast method after a standard dose (0.1 mmol/kg) *i.v*. bolus injection of gadopentetate dimeglumine (Gadovist) with a power injector at a rate of 6 cc/s, immediately followed by a bolus injection of 40 cc of an isotonic saline solution. Forty sequential image volumes containing 30 contiguous 3.5 mm slices were acquired every 1.9s using a PRESTO (principles of echo shifting with train of observation) multishot sequence during the first pass of the contrast bolus (TR/TE: 20/28.3 ms, flip angle = 7°, nominal voxel dimensions: 1.79 * 1.79 * 3.5 mm^3^).

### Data processing

Parametric maps of ADC, fractional anisotropy (FA), as well as axial and radial diffusivities were computed after correcting for image distortions using the MEDINRIA software (Toussaint et al. 2007). Parametric maps reflecting relative cerebral blood volume (rCBV), were performed on a remote station (Easy Vision). Inventory of the whole lesions developed during the 8 weeks of the study was performed using MIPAV software (Medical Image Processing And Visualization, downloadable from: http://mipav.cit.nih.gov/). Lesion volumetry was realized for each time point and for each active lesion with a semiautomatic method based on intensity thresholding. 3D enhanced T1-weighted images were used to achieve this inventory. All listed WM lesions were confirmed on 3D FLAIR images to rule out any enhancement artifacts or vasculature confounding.

Coregistration of 2D diffusion and perfusion images with the 3D T1 images, used as referential space, was computed using the “fusion” module of MEDINRIA software. Susceptibility artifacts observed on EPI sequence images were corrected by applying a nonlinear “thin plate spline transformation” (Davis et al. 1997).

## Results

All patients showed an increase in ADC in all active lesions. The transient ADC decrease during the initial gadolinium enhancement was only observed in two active lesions of one patient, a 32-year-old woman that presented a diagnosis of relapsed remitted (RR) defined by McDonald's criteria (McDonald et al. 2001). At inclusion time, she had an EDSS of 4, a Kurtzke pyramidal score of 2 and a Kurtzke sensory score of 2. The patient experienced 12 clinical relapses in the 5.4 years since her first symptom. Relapses consisted essentially in optic neuritis and recurrent sensitive disorders. She was treated with azathioprine, which was stopped in 2006, three years before the first MRI exam. No other immunoactive drugs or systemic corticosteroids were administered at the time of the study. During the MRI follow-up, the patient was asymptomatic, thus showed no clinical relapses despite the multiple lesions that appeared, and the lack of treatment.

Ninety-eight active lesions were identified in this patient during the period of 8 weeks. New enhancing lesions were detected at every time point. Average duration of enhancement was 2.24 weeks. Maximal number of enhancing lesions was observed at the 7th time point (Table 1). Thirty-six lesions with a volume larger than 10 mm^3^ were analyzed. Increased ADC was always observed at some point after gadolinium enhancement on T1-weighted images.

**Table 1:** Number and total volume (mm^3^) of enhanced lesions at each time point of the following

	Week 1	Week 2	Week 3	Week 4	Week 5	Week 6	Week 7	Week 8
Number	15	21	26	26	19	39	59	41
Total volume (mm^3^)	345.4	424.5	541.4	694.8	506.7	713.2	1635.0	1127.2

**Figure 1:**
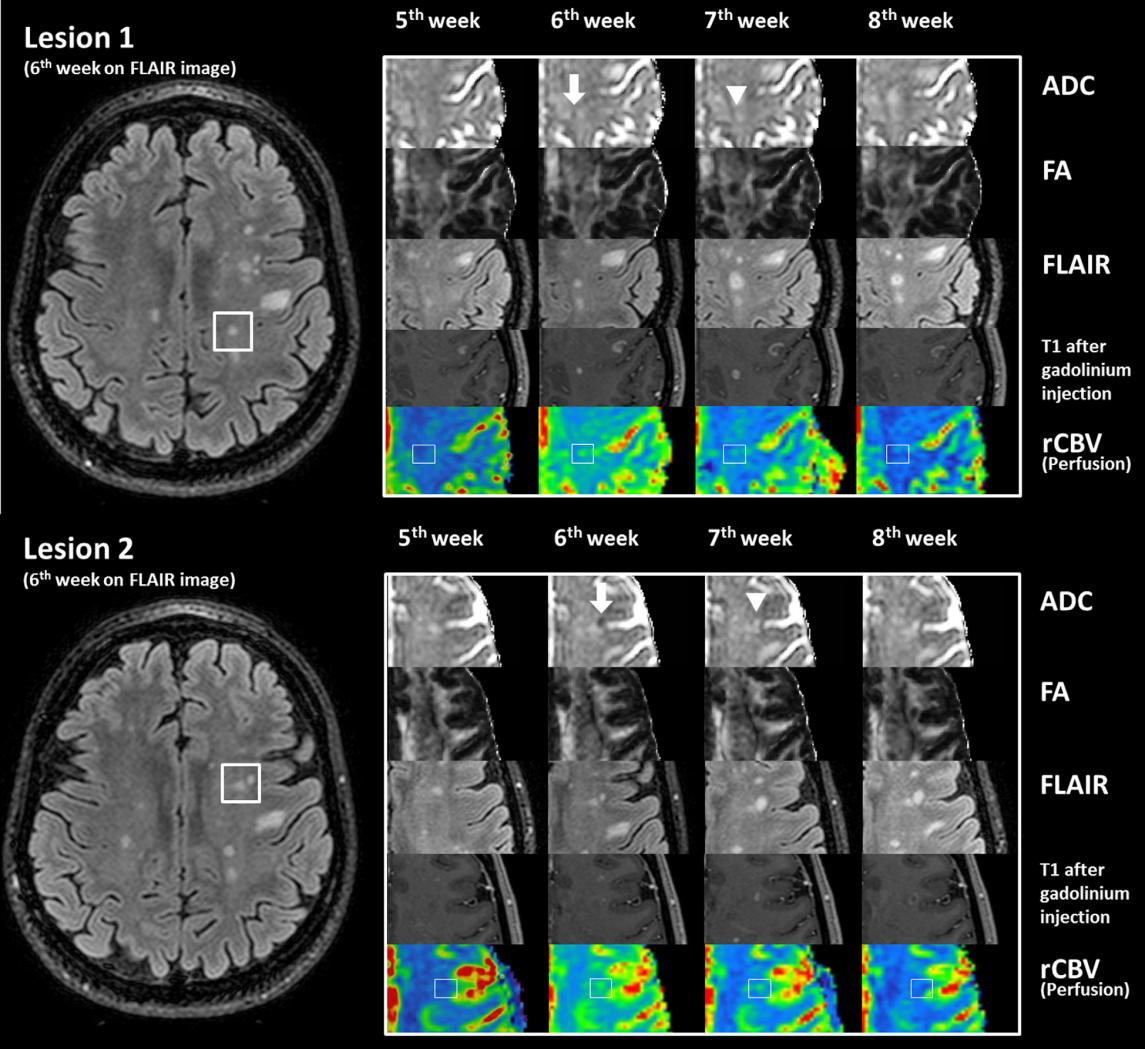
Weekly evolution of both active lesions using multimodal analysis: images of the active lesions at the last four weeks of the examinations seen on ADC and FA maps, FLAIR, T1 after gadolinium injection and rCBV images. Transient decreased ADC and higher rCBV values arised at the 6th week when lesion appeared with injected T1-weighted image (*white arrows*). ADC map of the following weeks shows a marked increase (*white arrow-head*).

In contrast, 2 of the 36 lesions, showed a transient decrease in ADC that was only observed at the time of first gadolinium enhancement (week 6). One week before this event (week 5), the mean ADC value in these two lesions was 2.31 × 10^−3^ mm^2^/s. On week 7, a marked increase in 48.9% and 34.7%, respectively, was observed. In both lesions, no significant changes in FA values occurred during the transient decrease in ADC (week 6). At week 7, FA drastically decreased of 44% for Lesion 1 and 43% for Lesion 2. However, rCBV values increased at the time of first enhancement and remained elevated for at least 2 weeks (Fig.1; Table 2).

**Table 2:** Diffusion and perfusion values of the lesions during the weekly MRI examinations

		Week 1	Week 2	Week 3	Week 4	Week 5	Week 6	Week 7	Week 8
Lesion 1	Volume (mm^3^)	–	–	–	–	–	4.39	50.98	78.22
	FA	0.36	0.42	0.43	0.40	0.39	0.36	0.20	0.20
	ADC (10^−3^ mm^2^/s)	2.32	2.34	2.31	2.32	2.27	**1.88**	2.80	3.13
	rCBV	0.69	0.96	0.85	0.85	0.68	**1.32**	**1.54**	0.62
Lesion 2	Volume (mm^3^)	–	–	–	–	–	5.27	68.55	137.64
	FA	0.37	0.38	0.37	0.39	0.39	0.39	0.22	0.27
	ADC (10^−3^ mm^2^/s)	2.38	2.35	2.24	2.25	2.34	**2.19**	2.95	2.84
	rCBV	1.19	0.97	1.00	1.04	1.13	**1.33**	**1.27**	**1.50**

Values in bold represent the transient decrease of ADC values, and the increase of rCBV values in both lesions.

## Discussion

Decreased diffusion in the context of acute MS lesion formation has been previously reported (Balashov and Lindzen 2012). However, the underlying physiopathologic mechanisms remain unclear. occurrence may be due to an inflammatory process leading to a local hypercellularity and/or an hypoxic/ischemic event inducing cytotoxic and vasogenic edema. In this multimodal weekly MRI follow-up, we had a unique chance to observe and analyze the evolution of diffusion and perfusion events occurring during the formation of two new enhancing lesions with a transient decrease in ADC values.

Vascular pathology, as an etiological contributor to the genesis of active MS lesions, was first suggested by histopathological findings reminiscent of alterations found in the early stages of white matter ischemia (Lassman 2003). As described by Lucchinetti et al., these findings correspond to the third immunohistochemical pattern of MS lesions, which is characterized by “distal dying back oligodendrogliopathy with oligodendrocytes apoptosis” (Lucchinetti et al. 2000). Further analogies of MS lesions with brain ischemia are the findings of “myelin associated glycoprotein” loss (Aboul-Enein et al. 2003), and the expression of hypoxia-inducible factor (HIF 1 alpha), an antigen identified as a hypoxic marker in active lesions (Semenza 2000). Taken together, these histological characteristics of pattern III lesions could be interpreted as outcomes of several pathological mechanisms. Firstly, vasogenic edema formation can induce passive microvascular compression with subsequent microischemic processes. Secondly, vessel walls can be affected by inflammatory reactions leading to vascular wall thickening and endovascular abnormalities with subsequent thrombus formation. In both cases, one could expect a decreased rCBV, reflective of ischemia. In contrast, the observed increase in rCBV is more consistent with reactive hyperemia in the context of hypercellular inflammation (perivascular cuffs) leading to decreased diffusivity. Indeed, the increase in cellular density confines the mobility of water protons in extracellular spaces. The diffusion restriction could also be affected by typical active MS lesions processes of perivenular tissue infiltration with macrophages and lymphocytes. Such ADC decrease has also been described in cerebral lymphoma (Cotton et al. 2006).

In this study, reduced diffusion was only seen at one time point in both lesions suggesting that the duration of this phenomenon is very short (in the range of 1–13 days) and appears only at the initial stage of active lesions. This finding is consistent with a rapid vascular/hypoxic event that could induce myelin energy metabolism alterations leading to cell swelling (cytotoxic edema) and lactate acidosis as frequently showed in active lesion by proton MR spectroscopy (Bhakoo and Pearce 2000). This mitochondrial metabolism dysfunction is consistent with the reduced diffusion observed by DTI (Qi et al. 2006).

In a similar protocol, Rocca et al. (2000) described 18 lesions followed with diffusion MRI at weekly intervals. All lesions demonstrated increased ADC, which is consistent with the low frequency of this phenomenon in our data set (2 out of the 36 lesions). Furthermore, the low frequency of this finding is likely to reflect the very short duration of this phenomenon, in the order of a few days or perhaps even just a few hours.

## Conclusion

We had a unique chance to observe and describe weekly diffusion and perfusion changes occurring during the formation of two new enhancing lesions characterized by a short initial decrease in ADC and delayed perfusion increase. The short delay (1 week) between MRI examinations allowed us to identify in two lesions new pathophysiological mechanisms in the genesis of plaque formation. The observed transient ADC decrease support the hypothesis of a short hypoxic event resulting probably from focal hypercellularity due to previous inflammation processes. This initial phase is followed by a reactional increased perfusion. These results suggest that the level of ischemic event may vary from lesion to lesion leading to different myelin damages and thus variable myelin reparation. We suggest that early ADC decrease could be an interesting marker of lesion genesis for the prognosis of lesion recovery.
